# Laboratory assessment of the anti-feeding effect for up to 12 months of a slow release deltamethrin collar (Scalibor®) against the sand fly *Phlebotomus perniciosus* in dogs

**DOI:** 10.1186/s13071-018-3094-z

**Published:** 2018-09-27

**Authors:** Samara Paulin, Régis Frénais, Emmanuel Thomas, Paul M. Baldwin

**Affiliations:** 1 MSD Animal Health Innovation SAS, 7 rue Olivier de Serres, CS 67131, 49071 Angers Technopole, Beaucouzé France; 2 MSD Animal Health Innovation GmbH, Zur Propstei, 55270 Schwabenheim, Germany

**Keywords:** Deltamethrin collar, Dog, Sand fly, Scalibor® protector band

## Abstract

**Background:**

Leishmaniosis/leishmaniasis consists of a wide group of diseases, caused by different *Leishmania* species and having different hosts. Leishmaniosis caused by *Leishmania infantum*, a disease primarily of dogs and humans, occurs after susceptible hosts are exposed to the feeding behavior of infected sand flies. A one-year laboratory study in dogs was designed to determine the 364-day anti-feeding efficacy of a slow release deltamethrin collar against the sand fly *P. perniciosus*, a common host of *L. infantum* in the Mediterranean basin.

**Methods:**

In this assessor-blinded study, 16 Beagle dogs were randomized into two groups using *P. perniciosus* engorgement rates from a Day -7 challenge. On Day 0, dogs in Group 1 received a placebo collar, while dogs in Group 2 received a deltamethrin collar (Scalibor® Protector Band). All dogs were caged, sedated and then exposed for 1 h to 85 (± 10) female and 15 (± 5) male *P. perniciosus* on Day 7 and every 28 days through Day 364. All flies, alive and dead, were aspirated from cages and from dogs, immediately counted and then frozen for assessment of blood engorgement. Anti-feeding efficacy was determined by comparing the arithmetic means of engorged female flies (alive, dead and moribund) in the deltamethrin group to the control group means. Insecticidal efficacy at the time flies were retrieved was assessed by comparisons between groups of mean live female fly counts.

**Results:**

In the deltamethrin group, relative to the control group, there was a significant reduction in arithmetic mean numbers of engorged *P. perniciosus* of 94–98% from Day 7 through Day 364. On Day 28, in the treated group relative to the control group, there was a 74% reduction in mean live fly counts, with between-group differences significant from Days 7 through 196, although insecticidal activity remained less than 50% from Day 56.

**Conclusion:**

Deltamethrin collar application to dogs reduced sand fly feeding by ≥ 94%, relative to unprotected control dogs, for 364 days. Thus, one collar applied to a dog can prevent or reduce the risk of sand fly transmission of *Leishmania* for one full year.

## Background

Leishmaniasis consists of a wide group of diseases, caused by different *Leishmania* species. Leishmaniasis caused by *Leishmania infantum*, a disease primarily of dogs and humans occurs after susceptible hosts are exposed to the feeding behavior of *L. infantum*-infected feeding sand flies. While many infections subclinical, in susceptible individuals clinical signs, attributed to protozoon-induced deposition of immune complexes, may develop soon after infection, or may be insidious in onset following an incubation period of months to years [1–3]. Poor body condition, lymphadenopathy and exfoliative dermatitis represent the most common clinical manifestations in dogs, followed by a range of signs including, ocular disease, epistaxis and lethargy, with a potentially fatal outcome [1–4]. Immunosuppressed individuals are at greatest risk of developing clinical leishmaniasis. Traditionally prevalent in warmer latitudes including southern Europe, northern Africa, Middle East and central Asia, South and Central America and sporadically in the USA, *Leishmania* is endemic in locations which favor the sand fly life-cycle [2, 4–7]. Non-arthropod modes of transmission have also been reported [1]. The northward geographical spread of *L. infantum* in Europe has been linked to climate change-induced increases in ambient temperatures and to increased travel of pets between endemic and non-endemic areas [2, 4, 8, 9]. Sand flies feeding on infected dogs, whether or not those dogs are presenting clinical signs, ingest the protozoon and then transmit infection to the host of their next blood meal, be it a dog, a human or another animal species. Prevention of sand fly feeding on dogs is therefore an essential feature of disease control that helps limit the occurrence of infection in dogs and in people.

Numerous publications have demonstrated that the pyrethroid deltamethrin, impregnated into a collar and worn by dogs during the sand fly exposure season, reduces the incidence of *Leishmania* seroconversion under natural challenge conditions [10–15]. While these field reports, as well as two small-scale laboratory-challenge studies [16, 17] suggest that the anti-feeding activity of this collar is season-long, there is a need for well-controlled studies to investigate the specific duration of the period over which the collar prevents sand fly feeding, and the degree to which feeding is prevented. A one-year study was therefore designed with the objective of determining the anti-feeding and insecticidal efficacy against *Phlebotomus perniciosus*, the most commonly occurring sand fly in the Mediterranean basin [5], provided by a single application of the deltamethrin collar to dogs.

## Methods

This was a randomized, assessor-blinded, single-site study in which the efficacy of the deltamethrin collar (Scalibor® protector band, Intervet International B.V., The Netherlands) in preventing engorgement of *P. perniciosus* in Beagles was compared to a placebo control group at the Charles River Laboratory in County Mayo, Ireland. Study personnel carrying out general health observations, clinical assessments, and assessments of *P. perniciosus* exposure, engorgement, and counting were masked to the treatments.

### Animal inclusion, randomization and treatment

Twenty Beagle dogs, individually identified by an implanted microchip, were acclimatized for eight days prior to the day of treatment. On Day -7, dogs were exposed to challenge with 60 ± 10 female and 10 ± 2 male, laboratory-bred adult *P. perniciosus* that were free of *Leishmania*. The 16 dogs (8 males and 8 females) with the highest sand fly engorgement rate were allocated to the study. Dogs were excluded if they had been treated with any product with insecticidal activity within three months prior to enrollment, or if there was evidence of skin disease on the dog’s neck at the intended site of application of the study product. The 16 selected dogs ranged in age from one to six years and on Day -8 weighed 9.8–13.4 kg.

The 16 qualifying dogs were ranked within sex from highest to lowest Day -7 sand fly blood engorgement rate and blocked. The first two females in each block of two dogs formed a replicate. Those in the first replicate were assigned to the two study groups (placebo group or treated group) using random order numbers derived from Fisher and Yates tables. This procedure continued until four females had been allocated to each group and was repeated for males until the 16 dogs (eight males, eight females) had been allocated to the two groups (*n* = 8 per group). An equal number of males and females were allocated per group.

Dogs assigned to Group 1 received a placebo collar; those assigned to Group 2 received a deltamethrin collar (Scalibor® protector band) at the label dose rate. The collars were placed on Day 0 and left in place for 365 days. Each collar was removed from the sachet directly before use. The length (not including buckle) and the weight of each collar were recorded and the collars were marked in permanent ink with each dog’s identification in case of accidental displacement during the study. The collar was fastened around the dog’s neck, adjusted until a comfortable fit was achieved so that two fingers could be inserted between the neck and the collar, and fastened. Excess collar was pulled through the buckle and any excess beyond 5 cm was clipped. To reduce the risk of collar displacement, a small cable tie was fastened at the end of the collar overlap and a second cable tie was fastened at the midpoint of the overlapping area.

### Animal husbandry

From the start of acclimatization (Day -8) to Day 6, dogs were pair-housed in indoor pens measuring approximately 2.0 × 2.0 × 2.4 m (length × width × height), with the exception of Days 0 and 1 when they were individually housed for treatment and to facilitate clinical assessments. From Day -3 until Day 6, dogs of the same sex were pair-housed within treatment groups, unless circumstances such as veterinary care required single housing. Due to an observation of pen-mate damage to collars during the first days of the treatment period, study dogs were individually housed (pens approximately 2.0 × 2.0 × 2.4 m) from Day 7 until the end of the study, the damaged collars having been replaced with a new collar. The anti-feeding and insecticidal results obtained on day 7 for the 4 dogs with damaged collars were excluded from the statistical analyses for day 7.

Each pen contained a resting area, and a small amount of wood shavings was used as bedding. Each single-housed dog was provided a toy and received social interaction for at least two minutes daily until Day 15 when interaction was extended to at least 10 min daily.

Dogs were fed a standard commercially available dog food once daily at the recommended rate (approximately 300 g/dog/day). Food was removed from each pen on the evening prior to each administration of sedative for sand fly exposure. Potable water was available *ad libitum via* stainless steel bowls in each pen, except during periods when animals were sedated for exposure to sand flies. Water bowls were removed prior to sedation and replaced after dogs had fully recovered from sedation. For the duration of the study, the temperature in the dog pens was maintained between 17–19 °C and the humidity ranged between 38–71%. Photoperiod was controlled for approximately 10 h light and 14 h darkness per 24 h period. Dogs were weighed on Days -8, 54, 82, 110, 145, 173, 208, 236, 264, 299, 327 and 362, and examined by a veterinarian on Days -8, 54, 166, 222, 278 and 362. Daily general health observations were carried out on all dogs by a trained technician, except on Day 0 when multiple clinical assessments were performed.

### Sand fly infestations and assessments

All sand flies, free of *Leishmania*, were obtained from a colony maintained since 2007 at Charles University in Prague, Faculty of Science, Department of Parasitology, Czech Republic. After receipt at the research facility the flies were maintained in the dark at 25 ± 3 °C, and at least 70% humidity.

Each dog was exposed to challenge with viable *P. perniciosus* on Days -7, 7, 28, 56, 84, 112, 140, 168, 196, 224, 252, 280, 308, 336 and 364. For the challenge procedure dogs were sedated with intramuscular medetomidine hydrochloride (1 mg/ml) which was reversed post-challenge by administration of intramuscular atipamezole hydrochloride (5 mg/ml). Sedated dogs were placed in an exposure chamber (approximate dimensions 0.6 × 0.6 × 0.9 m) in a room where temperature ranged between 22.7–28.6 °C and relative humidity ranged between 64–91%. To preclude any possibility of active product cross-contamination between groups, the exposure chambers used for each of the two groups were in separate sections of the animal housing unit. After the dogs had been placed in an exposure chamber, a container containing 85 (± 10) female and, to help stimulate female feeding behavior, 15 (± 5) male *P. perniciosus* was put into the chamber. Because sand flies are nocturnal feeders, the lights were turned off immediately after the lid of the container was removed [6]. After approximately 60 min, the lights were turned on and all live sand flies were collected using an aspirator. Disposable liner placed on the floor of each chamber was replaced after each dog had been removed. Where necessary, each chamber was wiped dry between dogs. Once all live flies had been removed, each dog was checked for dead and feeding flies prior to and after removal from the exposure chamber. All live feeding flies were aspirated from the dog and collected into the same vented container as the other live flies that had been collected. All dead and moribund *P. perniciosus* (on the dog and in the chamber) were collected using forceps and placed into a separate vented container. Prior to evaluation of their engorgement status all live flies were killed by freezing. One container at a time, the collected *P. perniciosus* were poured into a Petri dish or onto a white background and the engorgement status for each determined by inspection and, as required, use of a stereomicroscope to detect traces of a blood meal. All female sand flies (live, moribund and dead) were categorized as engorged or un-engorged. A fly was considered moribund if it did not show progressive movement but retained leg movement or twitching. All male sand flies were removed prior to counts/assessments of engorgement status and were not included in efficacy assessments.

### Assessments and statistical methods

The experimental unit was the individual animal. Arithmetic and geometric mean engorged *P. perniciosus* counts were calculated for each group on each day of counting and used to calculate the percent anti-feeding efficacy. Anti-feeding efficacy was determined by comparing the number of engorged female sand flies in the deltamethrin collar group to the mean number in the placebo control group at each post-treatment assessment. Anti-feeding counts included live, moribund and dead engorged flies. An effective dose was expected to provide a > 80% reduction in engorged *P. perniciosus* counts (based on arithmetic means) compared to the control group (as per EU Guideline 7AE17a).

Insecticidal efficacy was determined by comparing the mean counts of live female *P. perniciosus* (engorged + unengorged) in the treated group *versus* those of the placebo control group at the end of exposure, for each timepoint after treatment.

For each end-point geometric means were also calculated. The formula for determining efficacy was:

$$ \mathrm{Efficacy}\ \left(\%\right)=\left[\left(\mathrm{Mc}-\mathrm{Mt}\right)/\mathrm{Mc}\right]\ast 100 $$

where Mc is the arithmetic/ geometric mean count of Group 1 (placebo control), and Mt is the arithmetic/geometric mean count of Group 2 (deltamethrin collar group).

The groups were compared at each time point by a one-way ANOVA with a treatment effect on the sand fly data. The data were log-transformed if the normality and equal variance assumptions were not violated. Otherwise, the groups were compared by a non-parametric analysis using the Kruskall-Wallis test. All analyses were performed using SAS®/STAT (version 9.4). All statistical tests were two-tailed with a level of significance of 5%.

## Results

**Fig. 1:**
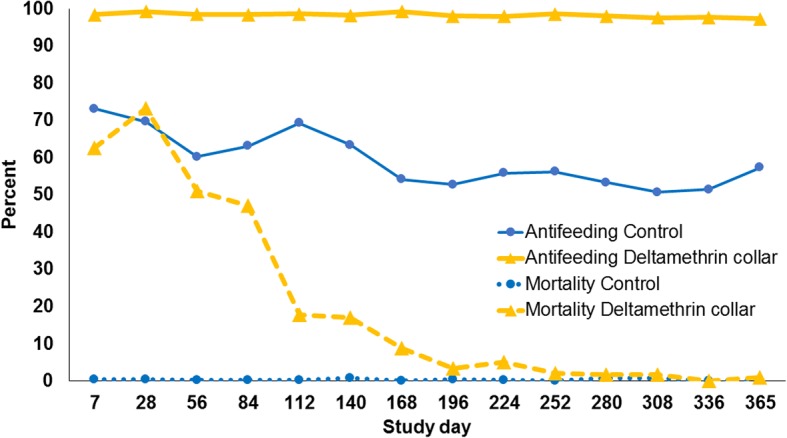
Anti-feeding and insecticidal efficacies in the deltamethrin collar treated group and the untreated control group. The between-group anti-feeding efficacy differences were significant at each assessment (*P* ≤ 0.0041)

There was a 97% reduction in arithmetic mean numbers of engorged *P. perniciosus* in the deltamethrin group, relative to the control group, at the first *P. perniciosus* challenge assessment, seven days post-application of the collars (Table 1, Fig. 1). The deltamethrin collar then provided sustained anti-feeding efficacy of ≥ 94% throughout the study. At each post-application assessment, the mean counts of engorged *P. perniciosus* in the deltamethrin group were statistically significantly lower than those in the control group (ANOVA or Kruskall-Wallis H-test: *P* ≤ 0.0041) (Table 1, Fig. 1). The maximum number of engorged *P. perniciosus* collected from any dog with a deltamethrin collar was four, while in the control group the minimum to maximum number of engorged sand flies ranged between 12–54, thereby validating the challenge methodology (Table 1).

**Table 1:** Mean numbers of engorged female sand flies and percent reductions in engorged sand flies on deltamethrin collar-treated dogs compared to untreated control dogs. Collars were placed on dogs on Day 0

Day	Control			Deltamethrin collar			Comparison		% Efficacy AM (GM)
	AM ± SD	GM	Range	AM ± SD	GM	Range	*χ* ^2^ *-value* ^b^ *F-*value^c^	*P*-value	
7^a^	22.4 ± 5.0	22.0	16–29	0.6 ± 0.5	0.5	0–1	*χ*^2^ = 8.2206, *df* = 1	0.0041	97 (98)
28	24.8 ± 9.7	23.2	14–41	0.6 ± 0.7	0.5	0–2	*χ*^2^ = 11.5315, *df* = 1	0.0007	97 (98)
56	33.9 ± 12.1	32.1	20–52	1.3 ± 1.8	0.8	0–5	*F*_(1, 14)_ = 113.92	<0.0001	96 (98)
84	31.5 ± 11.8	29.6	14–53	1.4 ± 1.1	1.1	0–3	*F*_(1, 14)_ = 135.63	<0.0001	96 (96)
112	25.4 ± 10.0	23.7	14–42	1.1 ± 1.4	0.8	0–4	F_(1, 14)_ = 111.83	<0.0001	96 (97)
140	31.6 ± 13.0	29.0	12–48	1.5 ± 1.4	1.1	0–3	*χ*^2^ = 11.4286, *df* = 1	0.0007	95 (96)
168	38.1 ± 8.7	37.3	27–53	0.6 ± 0.7	0.5	0–2	*χ*^2^ = 11.5315, *df* = 1	0.0007	98 (99)
196	40.0 ± 10.2	38.8	25–52	1.6 ± 1.4	1.3	0–4	*χ*^2^ = 11.3442, *df* = 1	0.0008	96 (97)
224	37.1 ± 6.3	36.7	28–46	1.8 ± 1.0	1.5	0–3	*χ*^2^ = 11.3947, *df* = 1	0.0007	95 (96)
252	36.4 ± 6.7	35.8	24–44	1.1 ± 0.8	1.0	0–2	*χ*^2^ = 11.4456, *df* = 1	0.0007	97 (97)
280	39.3 ± 5.9	38.9	31–51	1.6 ± 1.6	1.2	0–4	*χ*^2^ = 11.3947, *df* = 1	0.0007	96 (97)
308	41.4 ± 8.1	40.6	27–54	2.0 ± 1.2	1.7	0–4	*χ*^2^ = 11.4627, *df* = 1	0.0007	95 (96)
336	39.3 ± 8.9	38.3	25–52	1.9 ± 1.5	1.5	0–4	χ^2^ = 11.3442, *df* = 1	0.0008	95 (96)
364	35.4 ± 6.1	34.9	27–42	2.3 ± 1.3	2.0	0–4	χ^2^ = 11.3947, *df* = 1	0.0007	94 (94)

*Abbreviations*: *AM* arithmetic mean, *SD* standard deviation, *GM* geometric mean, *df* degrees of freedom

^a^Day 7 data for one control and three deltamethrin collar dogs were not included because their collars were damaged

^b^Kruskal-Wallis test

^c^ANOVA

Regarding the measurement of the insecticidal effect (Fig. 1) the deltamethrin collar treated dog group exhibited a significantly higher insecticidal effect between Day 7 and Day 196 (ANOVA or Kruskall-Wallis H-test: *P* ≤ 0.05), although this effect was below 50% after Day 56 through the remainder of the study (Table 2, Fig. 1). For both groups there were few moribund flies observed in the counts of live *P. perniciosus.*

**Table 2:** Mean counts of live and moribund female sand flies and percent reductions of sand flies on deltamethrin collar-treated dogs compared to untreated control dogs. Collars were placed on dogs on Day 0

Day	Control		Deltamethrin collar		Comparison		% Efficacy AM (GM)
	AM ± SD	GM	AM ± SD	GM	*χ* ^2^ *-value* ^b^ *F-*value^c^	*P*-value	
7^a^	82.3 ± 5.6	82.1	26.6 ± 9.6	25.2	*F*_(1, 10)_ = 182.0	<0.0001	69 (71)
28	81.1 ± 3.2	81.1	21.4 ± 10.1	18.9	*χ*^2^ = 11.3108, *df* = 1	0.0008	74 (77)
56	85.0 ± 2.4	85.0	40.6 ± 14.7	38.5	*χ*^2^ = 11.3947, *df* = 1	0.0007	56 (59)
84	85.3 ± 4.3	85.2	46.1 ± 9.5	45.2	*χ*^2^ = 11.3274, *df* = 1	0.0008	46 (47)
112	82.3 ± 3.2	82.2	67.4 ± 10.4	66.6	*F*_(1, 14)_ = 14.87	0.0017	18 (19)
140	85.8 ± 3.6	85.7	71.8 ± 14.4	70.2	*χ*^2^ = 6.1361, *df* = 1	0.0132	16 (18)
168	83.1 ± 2.0	83.1	75.8 ± 5.7	75.6	*χ*^2^ = 7.2357, *df* = 1	0.0071	9 (9)
196	84.3 ± 1.8	84.2	81.0 ± 4.3	80.9	*χ*^2^ = 4.4843, *df* = 1	0.0342	4 (4)
224	83.8 ± 1.5	83.7	80.4 ± 3.9	80.3	*χ*^2^ = 3.4436, *df* = 1	0.0635	4 (4)
252	83.0 ± 1.3	83.0	81.3 ± 6.6	81.0	*χ*^2^ = 0.1424, *df* = 1	0.7059	2 (2)
280	83.4 ± 1.6	83.4	82.4 ± 2.0	82.4	*F*_(1, 14)_ = 1.22>	0.2872	1 (1)
308	83.4 ± 2.8	83.3	81.3 ± 1.8	81.2	*F*_(1, 14)_ = 3.27	0.0922	3 (3)
336	80.9 ± 1.2	80.9	81.1 ± 1.5	81.1	*F*_(1, 14)_ = 0.14	0.7179	0 (0)
364	82.9 ± 2.4	82.8	82.1 ± 1.6	82.1	*F*_(1, 14)_ = 0.55	0.4724	1 (1)

*Abbreviations*: *AM* arithmetic mean, *SD* standard deviation, *GM* geometric mean, *df* degrees of freedom

^a^Day 7 data for one control and three deltamethrin collar dogs were not included because their collars were damaged

^b^Kruskal-Wallis test

^c^ANOVA

All dogs in both groups gained weight during the study. There were no treatment-related adverse events.

## Discussion

This is the first well-controlled laboratory study to directly demonstrate that the deltamethrin collar provides a strong and sustained anti-feeding effect on sand flies for up to one year. Therefore, a deltamethrin collar maintained on a dog for one year could prevent or reduce the risk of transmission of *L. infantum* for this extended period. These results help to explain the reported reductions in *L. infantum* infections from two-season field studies in which dogs received collars in consecutive years and were exposed to natural challenge [10–15].

In a study in northern Tunisia involving 80 *Leishmania*-free dogs, none of the 42 dogs treated with the deltamethrin collar seroconverted to *Leishmania*, while 15.8% of the control dogs seroconverted [10]. In another study, collars were applied to dogs in a town located in a *Leishmania* endemic region in southern Italy, while dogs in four neighbouring towns did not receive collars [11]. After the 1997 and 1998 seasons, 2.7 and 3.5% of collared dogs seroconverted, respectively, compared to a seroconversion rate of 5.4 and 25.8% of control dogs. This translated to protection rates provided by the collar of 50%, under conditions of apparent relatively low challenge, to 86% when challenge was more intense. In a 2008 report of a study in a coastal region of north-western Italy, just three of 119 (2.5%) collared dogs seroconverted, based on a *Leishmania* immunofluorescent antibody assay, compared with 15% of untreated control dogs [12]. A study in southern Italy estimated the anti-*Leishmania* protection rates provided by the collar, relative to un-collared control dogs, to be 72.3 and 41.2% in 2003 and 2004, respectively [13]. Of the seroconverting dogs in that study, clinical signs of canine leishmaniosis were significantly more frequent and rapidly progressive in control than in collared dogs. A study conducted in Brazil between January 2014 and November 2015 compared *Leishmania* infection rates in seronegative dogs from two neighboring endemic areas; dogs in one area received deltamethrin collars, those in the other area did not [15]. Despite the many limitations involved in an extended large-scale field study involving roaming domestic dogs, the results from serology testing showed a tendency for the deltamethrin collar to provide population-based protection against infection with *Leishmania*.

In another field study involving a twenty-year follow-up of canine leishmaniosis incidence in military working dogs, the reduction of the risk of leishmaniosis infection provided by deltamethrin-impregnated collars was demonstrated [14]. Usage of the deltamethrin collar started in 2002, and by comparing the incidence of leishmaniosis in the military kennels from 1993 to 2001, with the incidence observed in the period 2002 to 2012, the authors demonstrated a leishmanial protection of collared dogs of 93.8%.

Sand flies are active from dusk until dawn, with a reported peak of activity from 11 pm to 2 am [5]. The exposure period of one hour in our study was possibly shorter than exposures under field conditions. However, sedation of the study dogs would have made them more susceptible to becoming hosts for sand fly bites, as shown by the engorgement rates of flies exposed to control dogs. Although in the control dogs in our study those rates were lower than those reported from some other similar experiments, they were sufficient to validate the experimental methodology [18, 19]. Additionally, our results are consistent with, and extend those of a 1997 report of a shorter study from southern France [16]. In that study, the efficacy of the deltamethrin collar against sand fly bites in dogs sedated for two-hour exposure durations was 96% over 34 weeks.

Deltamethrin, like most pyrethroids, provides spatial-repellency and/or contact irritancy to insects [20, 21], and this effect is postulated to explain why the deltamethrin collar in our study provided a strong (≥ 94%) anti-feeding efficacy, with lower insecticidal efficacy (0–77%). Although the freezing of flies immediately following removal from the exposure chambers may have resulted in an underestimation of the insecticidal activity of the collar, the insecticidal activity we found generally aligns with an earlier study finding of a fly mortality between 21–60% over an 8-month exposure period of collared dogs to sand fly challenge [16]. We therefore postulate that the strong anti-feeding effect of the collar is due to avoidance of the sand flies to make sufficiently long contact with the protected dogs to engage in feeding behavior.

A number of studies have demonstrated that low volume insecticide “spot-on” and sprays applied to dogs can also be useful in the prevention of sand fly engorgement on dogs [19, 22–25]. Importantly, these products need to be applied once or twice monthly, meaning that owners must be conscientious in adhering to treatment recommendations. Additionally, optimal protection of on-dog, short-term measures requires accurate determination of the beginning and end of the sand fly season, which has been shown to vary by location, and from year to year due to changing climatic factors [5]. The extruded collar formulation used in this study provides extended deltamethrin release and is a key factor in the observed 12 month duration of anti-feeding efficacy. This extended duration of protection that could allow just once yearly treatment can be particularly relevant in geographies, for instance in areas of South America, where phlebotomine flies present an almost year-long risk of transmission of *L. infantum* [26, 27]. Additionally, recent evaluation of long-acting ectoparasiticides has shown that increasing the duration of protection leads to increased owner compliance and therefore more months of protection during the year [28]. Thus, once-yearly application of the deltamethrin collar to dogs reduces the risk of infection with *Leishmania*, relieves the pressure on dog owners for compliance to a repeated treatment scheme, and avoids the need for defining the beginning and end of the sand fly season.

## Conclusions

In dogs receiving the slow release deltamethrin collar, sand fly anti-feeding efficacy was ≥ 94%, relative to unprotected control dogs for one full year following treatment. Thus, one collar applied to a dog for one year can prevent or reduce sand fly transmission of *Leishmania* for this entire time.

## Abbreviations

AM: Arithmetic mean
EMA: European Medicines Agency
GM: Geometric mean
Mc: Mean count of Group 1 (placebo control)
Mt: Mean count of Group 2 (deltamethrin collar group)
SD: Standard deviation
VICH: International cooperation on harmonization of technical requirements for registration of veterinary medicinal products
